# Cerebral Ischemic Postconditioning Plays a Neuroprotective Role through Regulation of Central and Peripheral Glutamate

**DOI:** 10.1155/2018/6316059

**Published:** 2018-07-19

**Authors:** Jiulin You, Liangshu Feng, Meiying Xin, Di Ma, Jiachun Feng

**Affiliations:** Department of Neurology and Neuroscience Center, First Hospital of Jilin University, Changchun 130021, China

## Abstract

Following cerebral ischemia/reperfusion (I/R) injury, a series of pathophysiological processes are stimulated in both the central nervous system (CNS) and the periphery, including, but not limited to, the peripheral immune and endocrine systems and underregulation of the neuroendocrine-immune network. Glutamate (Glu) is an important excitatory neurotransmitter in the CNS; its excitotoxicity following cerebral ischemia has been a focus of study for several decades. In addition, as a novel immunoregulator, Glu also regulates immune activity in both the CNS and periphery and may connect the CNS and periphery through regulation of the neuroendocrine-immune network. Ischemic postconditioning (IPostC) is powerful and activates various endogenous neuroprotective mechanisms following cerebral I/R, but only a few studies have focused on the mechanisms associated with Glu to date. Given that Glu plays an important and complex pathophysiological role, the understanding of Glu-related mechanisms of IPostC is an interesting area of research, which we review here.

## 1. Introduction

Following a period of cerebral ischemia, ischemic postconditioning (IPostC), by applying a transient blood reperfusion/reocclusion series at the cerebral blood vessels, stimulates a variety of endogenous neuroprotective mechanisms and acts to reduce the effects of ischemia/reperfusion (I/R) injury [1]. However, clinical application of IPostC is limited due to the risk of additional ischemia to the brain. In recent years, the concept of conventional IPostC has been extended to include remote ischemic postconditioning (RIPostC), for example, in the distal limb [2]. IPostC activates a variety of endogenous mechanisms including the improvement of cerebral blood flow (CBF) during reperfusion, attenuated reactive oxygen species (ROS) production, inhibited local inflammation, and regulation of phosphorylation of multiple pathways including the PKC pathway, MAPK pathway, and the PI3K/Akt pathway [2], in order to exert neuroprotective effects.

Glutamate (Glu) levels in brain tissue and peripheral blood increase significantly following cerebral I/R injury [3, 4], and there are a variety of complex mechanisms underlying the interaction between CNS and the peripheral blood or organs. Glu not only exerts excitotoxicity and leads to neuronal death after ischemic stroke, but also acts as an immunoregulator to regulate multiple immune cells, which have invaded the central nervous system (CNS) or remain in the peripheral blood, resulting in the regulation of poststroke immune status.

Given that Glu plays an important and complex pathophysiological role after cerebral I/R injury, and among the various endogenous protective mechanisms stimulated by IPostC, there have been few systematic analyses and discussions about the mechanisms associated with Glu; here we sought to critically analyze the topic.

## 2. Ischemic Postconditioning Attenuates Both Central and Peripheral Glu Levels following Ischemia/Reperfusion

Glu, an important excitatory neurotransmitter in the CNS, is maintained at a low extracellular level under normal physiological conditions [5]. Under pathological conditions such as ischemic stroke, the accumulation of extracellular Glu stimulates NMDA receptors on neuron and glial cells, leading to the collapse of electrochemical gradient, activation of protein kinases and endonuclease, and degradation of important substances, and accelerates cell death through multiple pathways, in a process known as “excitotoxicity”. The maintenance of low extracellular Glu concentration depends on the Glu-glutamine cycle. Neuronal intracellular Glu, which is stored in presynaptic vesicles, is generated by the hydrolysis of glutamine (Gln) under the catalysis of glutaminase (Gls) and can be released to the extracellular fluid under certain physiological stimuli. To a large extent, the maintenance of extracellular Glu level depends on the effects of excitatory amino acid transporters (EAAT) on the astrocyte membrane, such as EAAT1 or EAAT2 [6], through which extracellular Glu can be transported into astrocytes. Subsequently the Glu in astrocytes can be used in several ways: by participating in the TCA cycle, the synthesis of glutathione (GSH), or the generation of glutamine under the catalysis of glutamine synthetase (GS). Glutamine is further transferred to extracellular space via SNAT 3/5 [7], then taken up via SNAT1/2 [8] in neurons, and hydrolyzed to Glu, which is then stored in vesicles. However, I/R breaks the steady state of this cycle.

As for the central nervous system (CNS), extracellular Glu levels rise sharply within a short period of time during acute ischemia and then drop immediately after reperfusion, though not returning to preischemia levels [9]. Consistent with this, some studies have suggested that the extracellular Glu concentration after reperfusion drops to its original level; this difference may be caused by factors such as experimental model differences, instrument accuracy, animal species, and ischemic severity [10]. During acute ischemia, (i) vesicular release from neurons constitutes a crucial component of extracellular Glu increase [11]; (ii) the expression of Glu transporter EAAT1 or EAAT2 decreases rapidly after ischemia and hypoxia, leading to ineffective removal of extracellular Glu [12, 13]; severe ischemia even leads to reverse transport of Glu via EAATs. After reperfusion, the coexistence of accumulation and elimination of extracellular Glu can occur via the following: (i) Glu can be partially removed by reconstructed blood flow [14–16]; (ii) the recovery of GS activity accelerates Glu utilization [17]; however, (iii) remaining dysfunction of EAAT1 or EAAT2 inhibits Glu uptake, contributing to extracellular accumulation; (iv) what is more, immune cells from reconstructed blood flow which have migrated to the CNS can also release Glu and exert neurotoxic effects [18–20].

As for peripheral blood, I/R also leads to increased Glu levels [3, 4, 21, 22], which is associated with ischemic severity and poor prognosis [3]. The mechanism underlying elevated peripheral Glu is not completely understood but may be related to the clearance of cerebral Glu. Moreover, during the ischemia phase, blood cell hypoxia may also result in the release of Glu. Hypoxia activates several immune cells in blood including neutrophils and T cells [23, 24], which release Glu after activation [25, 26]. For example, hypoxia-inducible factor (HIF), an important regulator of cellular metabolism, stimulates metabolic reprogramming in CD4+T cells and CD8+T cells, resulting in T cell activation [24]; hypoxia also stimulates the activation of neutrophils [23]. Activated neutrophils and T cells are able to release Glu, which may contribute to the elevated peripheral Glu. The accumulation of peripheral Glu is an unfavorable factor for the prognosis of cerebral ischemia and may be related to the production of ROS [4] through the “oxidative pathway” [27].

However, IPostC is able to exert neuroprotective effects by attenuating both the central and peripheral Glu level [3, 28–30], which will be further discussed below.

## 3. Ischemic Postconditioning Attenuates Central Glu Levels and Exerts Neuroprotective Effects

Since the timing of IPostC is the reperfusion phase, we will mainly focus on this period. Bonova P et al. reported that Glu in brain homogenates increased significantly after reperfusion. Surprisingly, IPostC reduces Glu in the homogenate to control levels and even significantly lower levels than the control on the first day following reperfusion [3]. However, its influence on extracellular Glu level has not been directly confirmed, probably due to the rapid decrease of extracellular Glu after reperfusion and the limitation of current detection methods. However, several studies have suggested that IPostC accelerates extracellular Glu clearance via a variety of mechanisms [3, 28–30].

### 3.1. Ischemic Postconditioning Upregulates EAAT2 Expression

Zhang W et al. performed 10-minute ischemia on neurons in the CA1 region, with most neurons observed to be dead after 72 hours. However, IPostC (6 cycles of 10 seconds/10 seconds of reperfusion/reocclusion) significantly reduced neuronal death. Furthermore, the Glu transporter EAAT2 was found to be significantly upregulated after 3, 6, 24, and 72 hours of reperfusion, which suggests that IPostC reduces cell death and that upregulation of EAAT2 expression may play an important role in this effect [28].

### 3.2. Ischemic Postconditioning Upregulates GS Level

Glutamine synthetase (GS) is an enzyme that is expressed in glial cells and accelerates extracellular Glu clearance by catalyzing Glu to generate glutamine and may attenuate Glu excitotoxicity. Zhang W and colleagues induced global ischemia in rats and performed 6 cycles of 10 seconds/10 seconds of reperfusion/reocclusion IPostC before final reperfusion. IPostC reduced neuronal death and resulted in a significant increase in GS expression compared with the sham group and the I/R group, indicating that the upregulation of GS expression after ischemia constitutes a neuroprotective mechanism [29].

### 3.3. Ischemic Postconditioning Attenuates the Infiltration of Immune Cells into the Central Nervous System, Inhibiting Its Glu Excitotoxicity

During inflammation, peripheral immune cells including monocytes and lymphocytes are recruited to the ischemic brain, contributing to further brain injury via Glu neurotoxicity, while IPostC attenuates the infiltration of immune cells.

Activated mononuclear cells in peripheral blood are able to infiltrate in the brain, release Glu, and contribute to neurotoxicity by activating NMDA receptors expressed by surrounding neuronal cells [18, 19]. Removal of Glu generated from activated monocytes/macrophages by the application of Glu decarboxylase attenuates its toxicity to human NT-2 cells by 12-13% [20]. After reperfusion, peripheral monocytes are recruited to the ischemic brain contributing to increased monocyte-derived macrophage number in the brain and decreased monocyte number in peripheral blood, while IPostC suppresses this trend and attenuates monocyte-derived macrophage numbers in the ischemic brain [31], which may release Glu participating in neurotoxicity. Therefore, the neuroprotective effect of IPostC may to some extent result from the inhibition of the accumulation of macrophages in brain to reduce the neurotoxicity of Glu.

In CNS, T cell derived TNF-*α* impairs the Glu clearance capacity of astrocytes by attenuating EAAT2, providing a pathogenic link to Glu excitotoxicity [32]. Moreover, activated CD8+ T cells, which infiltrate the brain directly, release Glu, causing damage to neurons, oligodendrocytes, and astrocytes. Following the activation of TCR on the surface of CD8+ T cells, (i) more Glu is generated through upregulation of glutaminase; (ii) more Glu is released through upregulation of vesicular proton-ATPase and vesicular Glu transporters required for filling vesicles with Glu. Subsequently, CD8+ T cells release Glu in a strictly stimulus-dependent manner [26] and then contribute to excitotoxic death of neurons in gray matter. Based on this finding, Glu release may represent a crucial effector pathway for CD8+ T cells, contributing to excitotoxicity in CNS inflammation. IPostC leads to the inhibition of T cell infiltration to the brain [31], contributing to the reduction of Glu excitotoxicity of T cells.

### 3.4. Ischemic Postconditioning Accelerates CNS Glu Clearance by Attenuating Peripheral Glu Levels

Peripheral blood Glu levels can, to a certain extent, regulate extracellular Glu levels in the CNS. This is due to the Glu concentration gradient between cerebral vascular endothelial cells and peripheral blood causing brain-to-blood Glu efflux. Extracellular Glu is transported via Na+-dependent transporters located on the antiluminal membrane and accumulates in endothelial cells. When its concentration exceeds that in plasma, Glu is transported across the luminal membrane into blood in a facilitative way [14]. As a result, decreased blood Glu level, which extends the concentration gradient between endothelial cells and the blood, accelerates the clearance of extracellular Glu in the brain and plays a neuroprotective role. In support of this, a large number of studies have demonstrated [15, 16] that the removal of Glu in blood by drugs results in an accelerated brain-to-blood Glu efflux, decreases infarcted brain volume and edema, and improves neurological outcome and mortality.

IPostC inhibits the increase of Glu level in peripheral blood after ischemia. Bonova P et al. reported that ischemia leads to meaningful elevation of blood Glu, while IPostC activates mechanisms resulting in rapid elimination of Glu in the circulatory system that could otherwise impede brain-to-blood Glu efflux mechanisms [3].

## 4. Ischemic Postconditioning May Regulate Immune Status after Ischemia/Reperfusion by Decreasing Peripheral Glu Level

I/R results in elevated levels of Glu in the blood, including whole blood, serum, and plasma [3, 4, 21, 22], while IPostC inhibits the increase of blood Glu. Bonova P et al. performed IPostC on cerebral I/R rats and collected whole blood samples [3]. It was found that IPostC leads to significantly decreased whole blood Glu compared to the I/R group. As for Glu levels in plasma or serum, this is yet to be investigated and should provide meaningful insight in future studies. How IPostC attenuates peripheral Glu levels is currently unknown but may result from decreased CNS Glu outflow.

Immunity and inflammation are important components of postischemia pathology.

First, in CNS, excessive Glu released in the early phase of ischemia activates resident immune cells in the brain, resulting in the generation of inflammatory mediators [33], activating innate and adaptive immunity. Shortly after Glu excitotoxicity, rapid changes of the microglial phenotype occur due to microglia activation. The activation and migration of microglia and production of cytotoxic mediators, chemokines, and cytokines such as TNF-*α* and IL-1*β* trigger inflammatory reactions after cerebral ischemia, further exacerbating brain damage [34]. Further, inflammatory mediators and oxidative stress promote the expression of adhesion molecules on cerebral endothelial cells, enhance blood-brain barrier (BBB) permeability, and accelerate the migration of peripheral leukocytes (neutrophils, lymphocytes, and monocytes) to the ischemic brain [16], resulting in the inflammatory reaction in CNS. However, peripheral immunity is depressed, leading to increased infection risk of peripheral organs such as the lung and urinary tract. Stroke-induced immunodepression results in pneumonia and is a major cause of delayed mortality in stroke patients [35, 36].

Glu is a novel immunoregulator of functions in several immune cells. Glu receptor (GluR) is widely present on the surface of innate immune cells (microglia, mononuclear cells, neutrophils, etc.) and adaptive immune cells (lymphocytes). GluR can be divided into iGluR (ionotropic Glu receptor) and mGluR (metabolized Glu receptor). In addition, there are three types of iGluR: NMDA receptor, AMPA receptor, and KA receptor. There are eight types of mGluR, mGluR1–8, which are further divided into three groups: group I (including mGlu1R and mGlu5R), group II (including mGlu2R and mGlu3R), and group III (including mGlu4R, mGlu6R, mGlu7R, and mGlu8R) [28]. Each kind of receptor has a different sensitivity and response to Glu [37, 38]. Glu regulates the immune state of immune cells (lymphocytes, neutrophils, mononuclear cells, macrophages, etc.) whether in central or peripheral systems; correspondingly, immune cells can have an effect on nearby cells or themselves via Glu release, participating in the development of pathological processes. It would be worth further exploring whether IPostC modulates poststroke immunity through Glu-related mechanisms.

### 4.1. Attenuated Peripheral Glu Level Downregulates Neutrophil Adhesion and Migration

After transient circulation (7-10 h), neutrophils leave the blood and migrate to tissues and organs due to the attraction of chemokines [39]. Under normal physiological conditions, there are few neutrophils in brain parenchyma. After cerebral ischemia/reperfusion, the number of neutrophils invading the brain increases significantly due to the presence of inflammatory factors, chemokines, and destruction of the BBB. Neutrophils invade the brain through the disrupted BBB or migrate into brain parenchyma following adhesion with endothelial cells, resulting in immune inflammation and further aggravated injury. Glutamate was found to be a novel chemokine that promotes neutrophil migration by the activation of type I mGluR [40]. Based on the report of Gupta R et al. [40], high concentrations of Glu in peripheral blood enhance neutrophil migration ability. Meanwhile, Glu-mediated NMDA receptor agonism increases the expression of CD11b [41], promotes adhesion, and “initiates” neutrophil activation [42]. Therefore, reduced Glu in peripheral blood is beneficial in order to attenuate the adhesion and migration of neutrophils, indirectly resulting in reduced brain damage. Based on these findings, it seems that IPostC is able to downregulate the number of central neutrophils by attenuating peripheral Glu level. Consistent with this inference, Liu SH et al. found that IPostC after myocardial I/R injury downregulates CD11b on neutrophils and reduces the adhesion activity of neutrophils [43].

### 4.2. Peripheral Glu and Lymphocyte Immune Status

Lymphocytes include T cells, B cells, and NK cells. Further, T cells can be classified into CD4+ Th cells and CD8+ cytotoxic Tc cells based on differential cell surface marker expression.

Various GluRs are expressed on the surface of T cells, including iGluR and mGluR. Glutamate can activate normal human T cells and is involved in the regulation of multiple T cell functions, including T cell adhesion, chemotactic migration, cytokine secretion, and gene expression. In addition, T cells can generate and release Glu and thus affect other cells nearby and themselves. GluRs are also expressed on the surface of B cells and participate in apoptosis and other activities [44]. However, among the existing studies involving Glu-related mechanisms, there have been more involving T cells. Few studies have focused on B cells and are mostly limited to mGluR; and the concentration of Glu involved is mostly at the level of millimolar, much higher than the physiological level. Thus, here we focus on T cells.

#### 4.2.1. Attenuated Peripheral Glu Levels Inhibit T Cell Adhesion

The immune effect of Glu is dose-dependent. However, whether central or peripheral, the decrease of Glu contributes to inhibited T cell migration towards CNS.

The concentration of Glu in cerebrospinal fluid in patients with progressive stroke is approximately 12.2 *μ*mol/L and in patients with stable infarcts is 3.8 *μ*mol/L, both of which are at low-level micromolar concentrations [45]. Low concentration of Glu in cerebrospinal fluid stimulates AMPA receptors on the surface of T cells: (i) inducing T cells adhesion and (ii) inducing T cell chemotactic migration towards key chemokines present in the CNS [46]. Plasma Glu concentration is much higher in patients with ischemic stroke; plasma Glu levels are usually between 100 and 400 *μ*mol/L. Glu at this concentration (i) induces T cell adhesion by iGluR-mediated increase of intracellular Ca^2+^ and (ii) promotes survival and function of activated T cells due to mGluR-mediated reduction of apoptosis [40]. Thus, reduced Glu levels after IPostC may result in downregulated T cell adhesion and attenuated T cell migration towards the CNS. Consistent with this inference, Zhao H et al. confirmed this phenomenon in mice [19].

#### 4.2.2. Peripheral Glu Regulates Circulating T Cell Activity

Rapid T-lymphopenia and long-lasting suppression of lymphocytic function have been observed in stroke patients [35]. Therefore, the maintenance of circulating T cell activity is important for preventing peripheral immunosuppression. The effect of blood Glu on circulatory T cells is two-sided. On the one hand, Glu can induce ROS generation in peripheral T cells, resulting in DNA breakage and cell injury [4]; on the other hand, Glu in the plasma of stroke patients (100-400 *μ*mol/L) can also inhibit T cell apoptosis through mGluR activation and promotes the survival and function of activated T cells [46].

The activity of T cells is affected by many factors; Glu is only one of them. The ultimate effect of Glu on peripheral T cells depends on the relative contribution of two seemingly opposite effects and the complex role of other factors after ischemia. Decrease of Glu, on the favorable side, is beneficial to reducing the mediated “oxidation pathway” and attenuating ROS generated by T cells, thereby alleviating DNA damage and helping prevent the loss of peripheral lymphocytic function.

#### 4.2.3. Glutamate May Be Involved in the Regulation of Th1/2 Immune Balance

T cells can be divided into CD4+ Th cells and CD8+ cytotoxic Tc cells based on different cell surface markers. Furthermore, CD4+ Th cells can be divided into Th1 and Th2 cells. Th1 cells secrete IFN-*γ* and IL-2, which promote CD8+Tc cell-mediated cytotoxicity and aggravate inflammation of the ischemic brain; Th2 cells mainly secrete IL-4, IL-5, IL-10, and IL −13, suppress local immune inflammation in CNS, promote peripheral humoral immunity, attenuate peripheral immunosuppression, and reduce the risk of infection of peripheral systems (such as the respiratory and urinary system) [46]. The balance of Th1/2 reflects the immune status of Th cells.

Whether central or peripheral, Glu shifts Th1/2 balance towards Th1, which contributes to the aggravation of both local inflammation in CNS and peripheral immunosuppression.

In the periphery, secretion of IL-10, an important anti-inflammatory factor generated by Th2 cells, whose main biological function is limitation and termination of inflammatory responses, can be reduced by peripheral blood Glu, resulting in prolonged and exacerbated inflammation [47]. This phenomenon indirectly suggests that Glu can inhibit Th2 immunoreactivity. Conversely, IFN-*γ* acts as a proinflammatory cytokine secreted by Th1 cells; secretion can be induced by NMDA receptor agonism, causing exacerbated inflammation [27].

In CNS, the microenvironment also modifies T cell polarization to Th1; Glu can promote this process. Glutamate accumulation that may result from impaired Glu uptake by astrocytes (e.g., after cerebral ischemia) strongly promotes Th1 production [48].

Whether IPostC affects Th1/2 immune balance and the Glu-related mechanisms involved provides a good target for further study. As mentioned earlier, Glu shifts the Th1/2 balance towards Th1, which is not conducive to prognosis. We deducted that IPostC downregulates Glu levels, which in turn reduces the proportion of Th1 cells. Consistent with this deduction, it has been reported that IPostC reduces the level of cytokines such as IL-1*β* and IL-6 in the ischemic brain [49]. Both IL-1*β* and IL-6 are cytokines that are secreted by Th1 cells. Therefore, an interesting field of research would be whether IPostC could inhibit the shift of Th1/2 immune balance towards Th1 by reducing Glu levels.

### 4.3. Ischemic Postconditioning Inhibits Central Inflammation and Attenuates Peripheral Immunosuppression—Possibly by Attenuating Peripheral Glu Level

Joo SP et al. found that IPostC suppresses central immune inflammation and attenuates peripheral immunosuppression in mice: immediately after 45 minutes of middle cerebral artery occlusion (MCAO) and before reperfusion, mice were treated with IPostC: 15 seconds of reperfusion/15 of seconds reocclusion, repeated for 3 cycles. It was found that the number of immune cells including monocytes/macrophages, CD4+ T cells, and CD8+ T cells decreased significantly in the brain, while it was increased in peripheral blood [31]. Moreover, Chen G et al. reported that RIPostC inhibits the activity of neutrophils in brain tissue.

As an immunoregulator, Glu may be involved in the mechanisms that underlie this phenomenon. As mentioned above, reduced peripheral Glu level after IPostC helps to attenuate the infiltration of circulating neutrophils towards the CNS and downregulate T cell adhesion, inhibiting the migration of CD4+ T cells and CD8+ T cells towards the ischemic brain. This helps to prevent the loss of peripheral immune cells, attenuates peripheral immunosuppression, and inhibits inflammation in the CNS. In addition, reduced Glu levels inhibit the “oxidation pathway” resulting in reduced ROS production, thereby mitigating lymphocyte DNA damage and then preventing the loss of lymphocytic function in blood [4].

However, there have only been a few studies focused on the influence of IPostC on Glu levels in peripheral blood, and the samples involved are whole blood samples. As for Glu levels in plasma or serum, we could not identify any appropriate studies. Given that Glu acts as a potential immune mediator and as an important biomarker in serum or plasma [21, 22], the effect of IPostC on peripheral Glu level and its relationship with immune status is worth further investigation.

## 5. Ischemic Postconditioning May Enable the Interaction between Neuroendocrine-Immune System and Glu

The CNS and periphery do not exist in isolation but affect each other in a mutual way: the CNS regulates peripheral endocrine and immune states by the neuroendocrine-immune system; correspondingly, peripheral immune states such as cytokines also activate CNS components such as the hippocampus and hypothalamus. IPostC regulates not only the CNS but also the periphery, and the role of neuroendocrine-immune regulatory networks may be involved in the underlying mechanisms.

### 5.1. Ischemic Postconditioning May Attenuate Peripheral Immunosuppression by the Inhibition of Sympathetic Nervous System Resulting from Reduced Glu Levels in Hippocampus and Hypothalamus

The sympathetic nervous system is a pathway through which the CNS and the immune system communicate with each other. For example, the presence of sympathetic nerve fibers in lymphoid organs and the release of norepinephrine from nerve terminals located in the direct vicinity of immune cells provide a mechanism by which norepinephrine might influence immune cell function. Released norepinephrine regulates the activity of immune cells in lymphoid organs. Correspondingly, products of activated immune cells might influence the activity of sympathetic nerves originating in the CNS, since circulating cytokines and cells are actively transported into the CNS. Upon activation, increased numbers of lymphocytes enter the CNS and produce cytokines and antibodies that can either protect against or contribute to several CNS pathologies [50].

As a source of stress, cerebral ischemia contributes to the activation of the sympathetic nervous system [51–53]. Kuriyama N et al. found that patients with a supratentorial acute stage cerebral infarction display a relative increase in sympathetic nerve output [51]. Consistent with this, the serum epinephrine and norepinephrine are elevated in patients after stroke. Meanwhile, the peripheral CD4+ T and CD8+ T cells are decreased [52], which suggests that excessive sympathetic adrenomedullar activation may be associated with immunosuppression. This was confirmed in vivo in rats: MCAO-induced spleen size reduction correlates with changes in epinephrine, norepinephrine, and cytokines. Blocking the sympathetic nervous system with propranolol can partly reverse immunodepression and reduction in spleen volume [53].

The sympathetic nervous system can be regulated by the hypothalamus and hippocampus. In addition, studies have shown that Glu in the hypothalamus and hippocampus results in increased sympathetic outflow [54–56]. Glutamate in the hippocampus stimulates norepinephrine release [54] and increases sympathetic outflow [55]. In addition, Glu in the hypothalamus contributes to sympathetic overactivation resulting in pathological processes [56].

Glutamate in the hippocampus and hypothalamus increases significantly in MCAO/reperfusion rats [57]. In addition, extracellular accumulation of Glu due to inhibition of EAAT2 leads to the activation of neuronal extrasynaptic NMDA receptors, resulting in increased parasympathetic neuronal activity and enhanced sympathetic outflow [58]. Therefore, the sympathetic overactivation may result from excessive Glu levels.

IPostC contributes to reduced Glu in the hippocampus [5] and upregulates EAAT2 levels [28], which may in turn inhibit sympathetic overactivation, further attenuating immunosuppression and inhibiting reduction of spleen size, though this remains to be directly confirmed. An interesting area of research combining Glu, sympathetic nervous system, and peripheral immune all together to explore the underlying mechanisms of IPostC would be useful.

### 5.2. Ischemic Postconditioning May Attenuate Glu Excitotoxicity by Regulating Thyroid Hormone Levels

The severity of I/R injury is closely related to thyroid hormone levels. Interestingly, different studies have shown different effects of thyroid hormone on the ischemic brain. Shuaib A et al. found that hypothyroidism protects the ischemic brain by reducing Glu release [59]; however, another study reported that exogenous administration of thyroid hormone during reperfusion phase exerts a neuroprotective effect, attenuating apoptosis and inflammation [60]. Though these results seem contradictory, we suggest that the effect of thyroid hormone may be time-dependent: during the ischemic phase, it may result in increased Glu release, aggravating brain injury, while during the reperfusion phase, it exerts a neuroprotective effect attenuating apoptosis and inflammation by accelerating the removal of Glu. Consistent with our hypothesis, exogenous administration of thyroid hormone exerted neuroprotective effects almost in all cases involving reperfusion phase [61, 62]. Thyroid hormone can eliminate excitotoxicity of Glu on astrocytes and protect the viability of cocultured neurons. This is because EAAT1 and EAAT2 are upregulated at both transcriptional and translational levels, thereby accelerating Glu uptake. In addition, thyroid hormone also nongenomically stimulates astrocyte Glu uptake, protecting hippocampal neurons against Glu excitotoxicity [62].

According to the above data, the timing of thyroid hormones exerting neuroprotective effects is the reperfusion phase, which is exactly the period when IPostC plays a role. It therefore seems that the underlying mechanism of IPostC has an interesting connection with thyroid hormone, although such studies have not been performed in cerebral I/R animal models but in myocardial I/R models. IPostC protects myocardium from I/R injury but fails to provide additional cardioprotection in a hypothyroidism rat model, with impaired thyroid hormone production [63].

Thyroid hormone levels in patients with ischemic stroke are reduced [64] and associated with a poorer functional prognosis to some extent [65]. Whether IPostC can increase the thyroxin levels and accelerate the clearance of Glu in CNS resulting in neuroprotection is worth further study.

### 5.3. Ischemic Postconditioning May Attenuate Peripheral Immunosuppression by the Inhibition of Hypothalamus-Pituitary-Adrenal Axis Resulting from Reduced Hypothalamic Glu Levels

In addition to the activation of sympathetic nervous system, the hypothalamus-pituitary-adrenal axis is also activated by ischemic stroke, leading to the release of cortisol from adrenal cortex. Cortisol levels are significantly increased in patients with ischemic stroke and are positively correlated with NIHSS scores. It is an independent prognostic marker for death and functional outcome [65]. Elevated cortisol can also result in peripheral immunosuppression, which is detrimental to prognosis.

Studies have shown that elevation of hypothalamic Glu levels results in increased activity of the hypothalamus-pituitary-adrenal axis [57]. After MCAO, Glu in hippocampus and hypothalamus increases rapidly [57] and peaks after 1 hour. Then rapidly decreases after reperfusion but increases again after 24 hours and remains at a high level. At the same time, corticotrophin-releasing hormone in hippocampus and hypothalamus is significantly increased with regard to mRNA level. During the peak period of injury, hypothalamus Glu content is positively correlated with corticotrophin-releasing hormone mRNA levels, accompanied by an increase in corticotrophin levels in plasma. Therefore, hypothalamus Glu may be the trigger factor resulting in overreaction of the hypothalamus-pituitary-adrenal axis [57], resulting in excessive cortisol release. Reduced hippocampus Glu induced by IPostC may attenuate the overreaction of hypothalamic-pituitary-adrenal axis, although this has not been confirmed directly and deserves further investigation.

### 5.4. Ischemic Postconditioning May Affect Immune Status by Attenuating Peripheral Glu and Then Regulate the Release of Cortisol, Which in Turn Affects Immune Status

The neuroendocrine-immune network is regulated mutually. On the one hand, the CNS regulates the immune and endocrine systems; on the other hand, the CNS is also affected by endocrine and immune systems. Interestingly the CNS initially affected by the immune system can once again regulate peripheral immune status via the neuroendocrine-immune system, forming a feedback loop.

Cortisol levels regulate immune status and are also regulated by the immune system. Proinflammatory cytokines, such as TNF-*α*, IL-1, and IL-6, mostly secreted by Th1 cells, not only participate in the CNS inflammation, but also activate the hypothalamus-pituitary-adrenocortical axis and increase cortisol levels in blood, resulting in decreased resistance to infectious diseases. Administration of the inhibitor of the interleukin-1 receptor (IL-1Ra) improves resistance to infectious diseases [66].

IPostC may inhibit the shift towards Th1 by downregulated peripheral Glu levels, resulting in attenuated proinflammatory cytokines secreted by Th1 cells, which may further inhibit the activation of hypothalamic-pituitary-adrenocortical axis, thus inhibiting cortisol release. Reduced cortisol levels affect the immune system once again, help attenuate peripheral immunosuppression, increase resistance to infectious diseases, and reduce delayed mortality due to poststroke infection.

## 6. The Underlying Mechanisms of Remote Ischemic Postconditioning May Be Related to Glu

Due to the risk of additional ischemia in the brain, conventional IPostC is limited in its clinical application; as a result of this, the concept of conventional IPostC has been extended to RIPostC, for example, transient ischemia in the remote limb [1, 2]. Researchers believe that this type of remote protection is associated with protective factors in the blood [2], such as exosomes, NO, and CO, which can enter the CNS via the blood.

### 6.1. Exosomes Derived from Remote Ischemic Postconditioning May Attenuate Glu Excitotoxicity in CNS

Exosomes participate in several important biological processes such as inflammation, immune response, and regulation of cell signaling pathways. Cells secrete exosomes and transport signal molecules to remote cells or organs resulting in their regulation and communication.

It has been reported that endothelial cell-derived exosomes directly protect nerve cells against I/R injury and are responsible for the protective role of remote IPostC in I/R injury [67, 68]. Kalani A et al. found that treatment with exosomes reduced NMDA receptor expression, infarct volume, and edema following I/R injury [68]. In addition, exosomes may provide protective effects against Glu excitotoxicity, which may be mediated by increased expression of the glutamate transporters, EAAT1 and EAAT2 [69]. In conclusion, exosomes derived from RIPostC may attenuate Glu excitotoxicity in I/R injury.

### 6.2. Remote Ischemic Postconditioning May Affect the Production of Gas Transmitters by Increasing Glu Levels near the Treated Site

In addition to conventional neurotransmitters such as acetylcholine, catecholamines, and Glu, some gas molecules in peripheral blood such as CO and NO have been studied as gas transmitters. The CO and NO provide neuroprotective effects against I/R injury by preserving BBB functional integrity, exhibiting potent antioxidant and antiapoptotic properties and increasing the recovery of CBF [70, 71].

NO and CO can be generated from endothelial cells. Glutamate can promote the generation of CO and NO by activating NMDA receptors on vascular endothelial cells [72, 73].

RIPostC is performed by a series of transient limb ischemia, while muscle ischemia results in increased Glu release. Liu Z et al. found that myocardial ischemia results in increased Glu release using a microdialysis method [74]. Consistent with this, intestinal I/R can also lead to increased Glu overflow [75]. Glutamate may be the key molecule in cellular ischemia. The increased Glu release resulting from limb ischemia can promote the generation of CO and NO by activating the NMDA receptors on vascular endothelial cells, exerting neuroprotective effects.

It has also been reported that there may be cross talk between NMDA receptors and nitrergic pathways in the myenteric plexus; I/R enhanced both Glu and NO spontaneous overflow from isolated ileal segments [75].

According to these data, RIPostC may upregulate the production of CO and NO near the treated site, which is then released into blood circulation and enters the CNS, exerting neuroprotective effects.

## 7. Ischemic Postconditioning May Improve Long-Term Neurological Outcomes after Stroke Possibly via Glutamatergic Mechanisms

The risk of neurodegenerative diseases and poststroke depression is greatly increased after cerebral ischemia. For example, at least one-third of stroke survivors suffer from depression [76]; the long-term consequences of forebrain ischemia include delayed Parkinson's syndrome [77]; further, seizure is a serious complication of stroke, indicating poor prognosis [78]. A major criticism of preclinical stroke studies is that they tend to focus too narrowly on histological reduction of acute infarct volume. Notably, a number of studies in human patients clearly demonstrated that lesion volume is of only subordinate importance to functional outcomes in the chronic phase after stroke [76]. Poststroke functional outcomes are more predominantly influenced by immune status, CNS neurotransmitter balance, and neuroendocrine system such as activity of the hypothalamus-pituitary-adrenal axis.

Therefore, the study of the neuroprotective effects of IPostC should not only be limited to the acute ischemic phase, but also include long-term neuroprotection. Although there are few relevant studies on long-term functional outcomes of IPostC after cerebral I/R, there are several studies on myocardial I/R. It has been reported that IPostC can improve long-term outcomes of myocardial I/R after 3.4 years, including improved left ventricular ejection fraction and myocardial perfusion grade, better epicardial and myocardial flow, and improvement in left ventricular function [79]. A preclinical study also suggested that repeated RIPostC after myocardial I/R protects against adverse left ventricular remodeling and improves survival in a rat model of myocardial infarction [80]. What is more, it has been reported that ischemic postconditioning inhibits renal fibrosis induced by ischemia-reperfusion injury in rats [81]. Moreover, in a clinical study, the application of brief repetitive remote ischemic preconditioning in bilateral arms for 300 days resulted in attenuated incidence of recurrent stroke, shortened average time to recovery (modified Rankin Scale score 0–1), and improved cerebral perfusion status (measured by SPECT and transcranial Doppler sonography) [82]. However, there are few studies focused on the long-term effects of IPostC on cerebral ischemia, which is an interesting direction for both preclinical and clinical research.

### 7.1. Ischemic Postconditioning May Attenuate Glu-Induced GAD Inhibition, Reducing the Risk of Delayed Parkinson's Syndrome and Poststroke Seizures

 Glutamate acts in important excitatory neurotransmission in the CNS, while *γ*-aminobutyric acid (GABA) acts in inhibitory neurotransmission. The Glu/GABA imbalance is an important inducing factor of several CNS diseases such as Parkinson's disease and seizures [77, 78]. An important enzyme regulating the balance of Glu/GABA is Glu decarboxylase (GAD). The synthesis of GABA is regulated through the activity of GAD, which catalyzes the synthesis of GABA from Glu.

The long-term consequences of forebrain ischemia include delayed Parkinson's syndrome. Parkinson's disease is associated with the progressive loss of dopaminergic (DAergic) neurons in substantia nigra (SN) and their axons projecting to striatum [77]. However, DAergic neurons are under GABAergic system control. GABAergic synaptic interactions with DAergic neurons are present in the SN; thus decrease of GABA in the SN will attenuate the amount of dopamine in the striatum. At least part of the cerebral impairment could be related to damage of the GABAergic system in striatum and SN. The delayed consequences of forebrain ischemia include decreased GAD level in SN [77], which leads to impaired synthesis of GABA from Glu.

The altered homeostasis of excitatory and inhibitory synaptic neurotransmission is also involved in poststroke seizures. The attenuated inhibitory synaptic neurotransmission by downregulated GABA contributes to the occurrence of seizure [83].

It has been reported that the damage of the GABAergic system is related to the decreased level of GAD, which results from the activation of NMDA receptors [84]. Glutamate-induced excitotoxic stimulation of NMDA receptor leads to decreased GAD protein levels of GABAergic neurons through intracellular calcium increase and protease activation including calpain and cathepsin. Thus upregulated extracellular Glu after stroke may result in decreased levels of GAD.

IPostC may attenuate Glu-induced inhibition of GAD in GABAergic neurons by downregulating CNS Glu level, reducing the risk of delayed Parkinson's syndrome and poststroke seizures.

### 7.2. Ischemic Postconditioning Regulates the Glu Level and Immune Status in CNS and May Reduce the Risk of Poststroke Suppression

The development of comorbid depression after stroke is clinically highly significant because poststroke depression is associated with increased mortality, slows recovery, and leads to worse functional outcomes.

Immune deregulation and, in particular, altered proinflammatory signaling have long been implicated in depression [76]. The hypothalamus-pituitary-adrenal axis is activated and blood cortisol levels increase after ischemic stroke. The elevation of corticoids also enhances immune function within the CNS, which is mediated by corticosterone-induced, overactivated NMDA receptor-mediated microglia activation [85]. Accordingly, IPostC attenuates the release of cortisol by inhibiting hypothalamic-pituitary-adrenal axis activity through glutamatergic mechanism, thereby reducing the risk of poststroke depression mediated by CNS inflammation.

The overactivated glutamatergic system and NMDA receptor agonism are associated with depressed mood, a reduction of the glutamatergic activity; that is, NMDA receptor antagonism might exert antidepressant effects [85]. NMDA antagonists such as MK-801 and ketamine exhibit antidepressant effects in different animal models. Slight antidepressant effects in humans have also been observed using NMDA receptor antagonists, amantadine and ketamine. Riluzole, an antiglutamatergic agent believed to increase glutamatergic uptake into astrocytes, is under intensive investigation for its antidepressant potential [86]. Besides, astrocytes are relatively few compared to activated microglia in patients with depression. Loss of astrocytes seems also to be associated with an impaired reuptake of Glu from the extracellular space into astrocytes by Glu transporters such as EAAT1/2. Impaired Glu reuptake from the extracellular space by astrocytes prolongs synaptic activation by Glu, which is associated with depressed mood.

It has been reported that IPostC significantly upregulates EAAT2 expression after I/R injury, which may attenuate extracellular Glu and inhibit glutamatergic activity, reducing the risk of poststroke suppression.

## 8. Conclusion

In summary, cerebral I/R results in elevated Glu levels both centrally and peripherally, which exerts deleterious effects, while IPostC attenuates central and peripheral Glu levels. However, the current research is limited, and the biological samples involved are mostly tissue samples or whole blood samples. As for the CNS, there is no direct evidence that IPostC attenuates Glu excitotoxicity; for peripheral systems, there has been no study focused on serum or plasma Glu levels. Therefore, whether and how IPostC attenuates central and peripheral Glu levels are worth further study.

**Figure 1:**
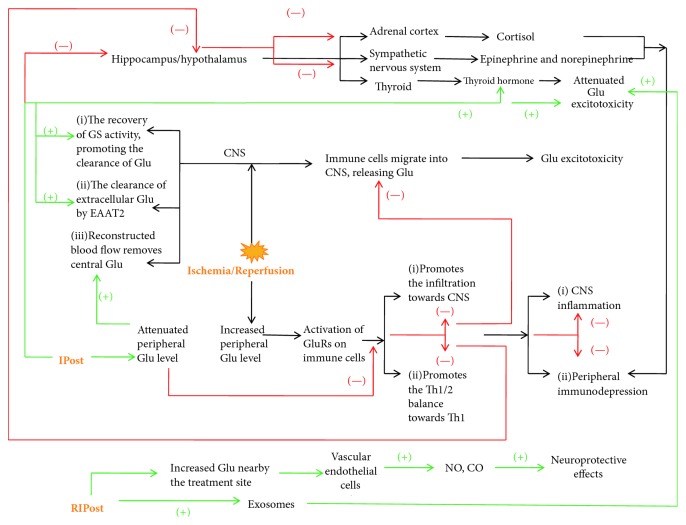
Glutamate-related mechanisms that may be associated with ischemic postconditioning.

Glutamate not only exerts an excitotoxic effect but also regulates immune reactions. The underlying mechanisms of suppressed central inflammation and attenuated peripheral immunosuppression resulting from IPostC may be associated with attenuated Glu. Although Glu may not be directly involved in the regulation of peripheral immune organs, such as the spleen, it may indirectly inhibit the size reduction by regulating sympathetic nerve activity. What is more, IPostC may connect the central and peripheral via Glu by the neuroendocrine-immune system, not only exerting a protective effect on the CNS, but also protecting the peripheral organs to resist poststroke infection (Figure 1).

Ischemia/reperfusion results in a series of pathophysiological changes both centrally and peripherally. IPostC is involved in the regulation of these pathophysiological changes. “(+)” refers to the promotion of certain changes and is marked in green; “(-)” refers to the suppression of certain changes and is marked in red. In conclusion, ischemia/reperfusion results in increased central and peripheral Glu. IPostC inhibits this increase and plays a protective role. With regard the CNS, (i) it promotes the expression of EAAT2 and accelerates the clearance of extracellular Glu; (ii) it upregulates the GS levels and accelerates the utilization of Glu; (iii) it accelerates the clearance of central Glu by reconstructed blood flow; (iv) it suppresses the migration of immune cells towards CNS and attenuates Glu excitotoxicity. For the periphery, by the downregulation of Glu levels, (i) it inhibits the migration of immune cell towards the brain, attenuates the loss of peripheral immune cells, and attenuates peripheral immunosuppression; (ii) it inhibits the shift of Th1/2 balance towards Th1. For the neuroendocrine-immune network, (i) it downregulates hippocampal and hypothalamic Glu, inhibits the overactivated sympathetic nervous system, and attenuates peripheral immunosuppression; (ii) it inhibits hypothalamic-pituitary-adrenal axis and attenuates peripheral immunosuppression; (iii) it inhibits the shift of Th1/2 balance towards Th1, reduces the secretion of proinflammatory factors, and attenuates the stimulation of the hypothalamus, resulting in reduced cortisol release.

As for RIPostC, (i) it promotes the production of exosomes and inhibits central Glu excitotoxicity; (ii) it may affect the local production of CO and NO near the treated site.

IPostC stands for ischemic postconditioning; RIPostC stands for remote ischemic postconditioning; CNS stands for central nervous system; GS stands for glutamine synthetase; EAAT stands for excitatory amino acid transporter; Glu stands for glutamate.

IPostC stimulates a variety of endogenous protective mechanisms. However, the understanding of Glu-related mechanisms is relatively poorly understood, presenting an interesting topic for research.

## Abbreviations

AMPA receptor: *α*-Amino-3-hydroxy-5-methyl-4-isoxazolepropionic acid receptor
BBB: Blood-brain barrier
CBF: Cerebral blood flow
CNS: Central nervous system
EAAT: Excitatory amino acid transporter
GABA: *γ*-Aminobutyric acid
GAD: Glu decarboxylase
Gln: Glutamine
Gls: Glutaminase
Glu: Glutamate
GluR: Glu receptor
GS: Glutamine synthetase
GSH: Glutathione
iGluR: Ionotropic Glu receptor
IPostC: Ischemic postconditioning
I/R: Ischemia/reperfusion
KA receptor: Kainite receptor
MCAO: Middle cerebral artery occlusion
mGluR: Metabolized Glu receptor
NMDA receptor: N-Methyl-D-aspartate receptor
RIPostC: Remote ischemic postconditioning
ROS: Reactive oxygen species
SN: Substantia nigra
SNC: Substantia nigra pars compacta.
